# Changing Safety Net Healthcare Systems to Age-Friendly Healthcare Systems: Lessons Learned

**DOI:** 10.1093/geroni/igaa057.2957

**Published:** 2020-12-16

**Authors:** Anna Faul, Nina Tumosa

## Abstract

Federally Qualified Health Centers (FQHC) are important safety net providers in disadvantaged communities. As outpatient clinics in these areas, they qualify for specific reimbursement systems under Medicare and Medicaid. Age-friendly health care is an urgent need to be able to provide quality healthcare to more than 46 million Americans age 65 and older, with that number projected to double to more than 98 million by 2060. Friendly healthcare systems require a focus on the 4Ms framework and is focused on improving the health of people at every life stage and in every community across the country. The 4Ms are as follows: 1) What Matters: Aligning care with each older adult’s specific health outcome goals and care preferences; 2) Medications: If medications are necessary, use age-friendly medications that do not interfere with What Matters, Mentation or Mobility; 3) Mentation: Prevent, identify, treat and manage depression, dementia and delirium across settings of care and 4) Mobility: Ensure that older adults move safely every day in order to maintain function and do What Matters. An age-friendly health system is one in which every older adult’s care is guided by these evidence-based practices (the 4Ms), where the care causes no harms, and where the care is consistent with what matters to older adults and their families. In this symposium five Geriatric Workforce Enhancement Programs at five diverse universities will share their experiences with supporting FQHC in their areas to become age-friendly healthcare systems. The unique lessons learned at these different sites will be shared.

